# The role of hormones in sepsis: an integrated overview with a focus on mitochondrial and immune cell dysfunction

**DOI:** 10.1042/CS20220709

**Published:** 2023-05-05

**Authors:** Miranda J. Melis, Muska Miller, Vera B.M. Peters, Mervyn Singer

**Affiliations:** Bloomsbury Institute of Intensive Care Medicine, Division of Medicine, University College London, London, UK

**Keywords:** hormones, immune response, mitochondria, organ dysfunction, sepsis, stress response

## Abstract

Sepsis is a dysregulated host response to infection that results in life-threatening organ dysfunction. Virtually every body system can be affected by this syndrome to greater or lesser extents. Gene transcription and downstream pathways are either up- or downregulated, albeit with considerable fluctuation over the course of the patient’s illness. This multi-system complexity contributes to a pathophysiology that remains to be fully elucidated. Consequentially, little progress has been made to date in developing new outcome-improving therapeutics. Endocrine alterations are well characterised in sepsis with variations in circulating blood levels and/or receptor resistance. However, little attention has been paid to an integrated view of how these hormonal changes impact upon the development of organ dysfunction and recovery. Here, we present a narrative review describing the impact of the altered endocrine system on mitochondrial dysfunction and immune suppression, two interlinked and key aspects of sepsis pathophysiology.

## Sepsis – definitions, clinical features, and pathophysiology

Sepsis is defined as a dysregulated host response to infection that leads to life-threatening organ dysfunction [1]. It can be triggered by a wide range of organisms, including bacterial, viral, fungal, parasitic or atypical, and presents in many different guises. While the focus of infection usually becomes apparent with disease progression, sepsis often presents with non-specific signs and symptoms that evolve into various combinations of organ dysfunction. This generally occurs over several days but, occasionally, within hours of initial symptomaticity. Sepsis is one of the commonest causes of death worldwide with overall mortality rates of approximately 15–20%. However, the risk of dying increases to over 40% in shocked patients [2]. The elderly, frail, and those with underlying comorbidities (e.g., cancer, immunosuppression, chronic organ failure), malnourishment and social deprivation are at much greater risk of both developing sepsis and dying as a consequence.

Most body organ systems are involved to greater or lesser degrees, including cardiovascular, respiratory, renal, hepatic, neurological, coagulation and immune systems. This can be variably manifest as differing clinical patterns – ‘subphenotypes’ [3] – with combinations of hypotension and poor peripheral perfusion due to vasculopathy ± cardiomyopathy, impaired gas exchange (termed ‘acute lung injury’ and, in its most severe form, ‘acute respiratory distress syndrome’), oligo-anuria and azotaemia (‘acute kidney injury’), hyperbilirubinaemia and coagulopathy from deranged liver function, an altered conscious state ranging from confusion through agitation, drowsiness and coma (septic encephalopathy), motor and sensory disturbances (neuromyopathy), and coagulopathy related to both depressed production and increased turnover of clotting factors and platelets. ‘Disseminated intravascular coagulation’ is often used as a descriptor of the coagulopathy, but this is usually a misnomer as intravascular clots with downstream infarction are rarely visualised either by imaging studies or at post-mortem. As described in more detail below, different components of the immune system are both over-activated and depressed. This fluctuates over time and drives both an exaggerated inflammatory response that triggers downstream organ dysfunction, as well as inducing immunosuppression that increases the patient's susceptibility to secondary infections.

### The inflammatory response

**Figure 1:**
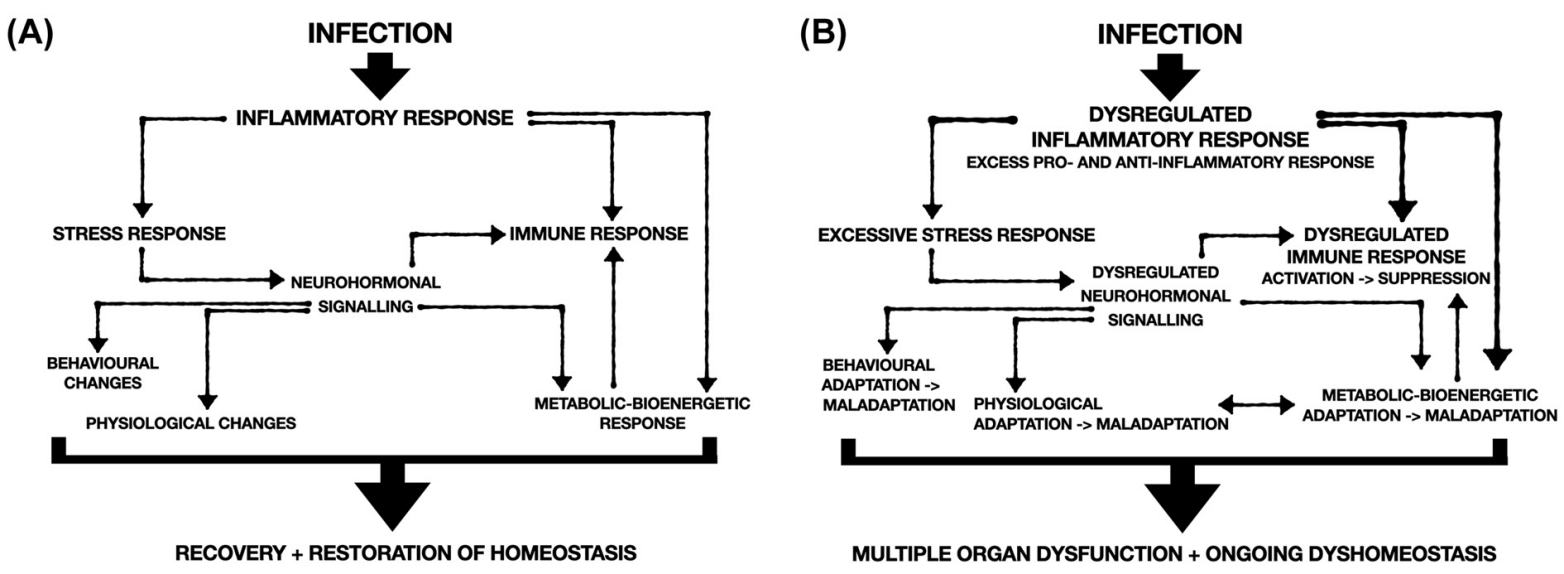
Appropriate (A) and inappropriate (B) host responses to an infectious insult resulting in either resolution of the infection or progression to multiple organ dysfunction Similar pathways are involved yet, for reasons still unclear, are dysregulated and exaggerated in the latter situation.

The pathophysiology of sepsis is still incompletely understood. There is a highly complex interaction between the host inflammatory response, neuro-hormonal signalling, and modifications in behaviour, physiology, bioenergetics, and metabolism. Similar pathways are involved in the appropriate host response to infection that enables the body to deal with the infectious illness yet without incurring the unwanted downstream sequelae of multi-organ dysfunction (Figure 1). Why some patients develop an inappropriate and dysregulated response is still unclear but likely involves multiple factors including genetic, epigenetic, ageing, comorbidities, environmental, and iatrogenic.

The initiation of sepsis is related to identification by specialised host receptors of pathogen-associated molecular patterns (PAMPs), i.e., pathogenic microorganisms or their constituents such as endotoxins, exotoxins and DNA. These pattern recognition receptors (PRRs) are located on innate immune cells (e.g. macrophages, monocytes, neutrophils and dendritic cells), endothelial and epithelial cells. They are either membrane-bound (e.g. the Toll-like receptor (TLR) system) or cytoplasmic (e.g. NOD-like receptors). PRRs can also be activated by damage or injury to host cells that release damage-associated molecular patterns (DAMPs) extracellularly and into the circulation. Examples of DAMPs include host DNA, RNA, mitochondria, and proteins such as heat shock proteins, HMGB1 and S100.

Activation of PRRs induces gene transcription leading to increased production and release of a wide range of both pro- and anti-inflammatory mediators including cytokines (e.g. tumour necrosis factors, interleukins, chemokines and interferons), eicosanoids and nitric oxide. Of note, as many, if not more, gene transcripts are down-regulated by the inflammatory process [4] and this varies both between organs and temporally [5]. There is a parallel activation and depression of pathways within the different body systems that is a characteristic of sepsis. As highlighted later, this equally applies to the endocrine system.

### Organ dysfunction: a metabolic-bioenergetic shutdown?

The outpouring of mediators have either direct or indirect downstream actions on endothelium, epithelium and organ-specific cells that modify organ function through changes in circulation and metabolism, including altered utilisation of substrate or oxygen. The circulation is modified by increased capillary leak, decreased vascular tone, heterogenous areas of vasodilatation and vasoconstriction within the microcirculation, and myocardial depression. The net result of these changes is altered perfusion and delivery of substrate and oxygen to tissues.

In tandem, there are metabolic and bioenergetic alterations. Early on in the septic process there is an increase in metabolic activity that is geared to fight the underlying infection. This energy expenditure needs to be fuelled by increased oxygen utilisation. However, with failure to promptly resolve the excessive inflammatory response and illness progression, there is a bioenergetic/metabolic switch with a downturn of body processes including muscular activity, anabolism and cell repair [6]. As discussed later, mitochondrial dysfunction appears to be a key player in triggering this metabolic shutdown.

We have previously argued this metabolic shutdown may represent a protective phenomenon in an oxygen/substrate limited environment [7]. This change in strategy shifts the focus towards cell survival which will enhance the possibility of ultimate recovery of the failed organs and the patient. Akin to hibernation or estivation, membrane integrity and ionic pumps are maintained at the expense of sacrificing normal energy-dependent cellular functions. This is manifest clinically and biochemically as organ dysfunction/failure. ‘Failure’ however carries a negative connotation. Organ shutdown may represent a temporary defensive tactic aimed at enabling subsequent renewal, especially in organs with poor regenerative capacity. Support for this hypothesis comes from the repeated demonstration of minimal cell death in organs taken from patients dying of sepsis [8–10]. While organ hypoperfusion at macro- and microcirculatory levels represents an important trigger of these downstream effects, this alone is insufficient to explain organ dysfunction in the absence of structural damage.

Mitochondria are present in all cells except erythrocytes. Other than their role as the predominant ATP generator in most cell types, they have important functions in regulating cell death and intracellular calcium, and are a major site of heat production and hormone production (e.g. cortisol). Mitochondria are the main utilisers of oxygen and producers of reactive oxygen species (ROS) within the body, and their activity and turnover (biogenesis) are influenced by multiple hormones. Mitochondrial dysfunction is well described in sepsis [11] and is implicated in failure affecting multiple organs including heart [12], kidney [13], liver [14] and brain [15]. The role in immune dysfunction is discussed below. Our group has previously described mitochondrial perturbations in patients [16,17], animal models [18–21], and in cell and tissue models [22,23].

## Immune (dys)regulation during sepsis: activation and suppression

Activation of the immune system by PAMPs and DAMPs aims to neutralise the pathogen yet excessive activation can result in tissue injury and can paradoxically render the host more vulnerable to subsequent infection, especially if the inflammatory state is both severe and prolonged [4,24,25].

As the pro-inflammatory response is mounted, the body simultaneously initiates a counterbalancing anti-inflammatory response, with the release of anti-inflammatory cytokines such as interleukin (IL)-1 receptor antagonist and IL-10 [26,27]. Combined with immune cell anergy and exhaustion, decreased chemotaxis, and increased apoptosis of peripheral blood mononuclear cells (PBMCs) and splenocytes [28], the net result is immunosuppression affecting both innate and adaptive immune systems and a failure to return to normal homeostasis. Consequently, the risk of secondary infection is enhanced by gut-derived Gram-negative organisms, opportunistic pathogens such as fungi, and reactivation of viruses such as cytomegalovirus that would rarely compromise a healthy host.

Anergy and exhaustion are produced by different mechanisms. Neutrophils show delayed apoptosis and a deficit in anti-microbial effector function, including oxidative burst capacity and chemotactic activity, while both neutrophils and PBMCs have a diminished cytokine and phagocytic response to *ex vivo* stimulation [29,30]. There is marked depletion of natural killer (NK) cells, CD4^+^ and CD8^+^ T-cells, and B-cells secondary to accelerated apoptosis [25,28], suppressed CD4^+^ T-helper (Th)1, Th2, and Th17 cell function [25], lower pro-inflammatory cytokine production and increased expression of checkpoint regulators such as programmed cell death-1 (PD-1). The density of cell surface receptors on circulating monocytes, macrophages and dendritic cells such as HLA-DR that present peptide antigens to the immune system are depleted. Dendritic cells also show increased apoptosis and IL-10 production [31]. Expansion of myeloid-derived suppressor cells contributes to decreased monocyte function, while the proportion of circulating immunosuppressive regulatory T-cells (Treg) also increases [32,33]. In the adaptive immune system B-lymphocytes are also depleted with reduced production of immunoglobulins [28,34].

The sum total is immunosuppression that can persist for weeks or even months after critical illness with an increased risk to the patient of secondary infection. This state of immunosuppression can contribute to poor longer-term outcomes. Up to 60% of critically ill survivors require subsequent rehospitalisation in the year following discharge, most often due to infection, and one-in-six die [35].

Although precise mechanisms underlying immune anergy, exhaustion and increased apoptosis remain to be elucidated, mitochondrial dysfunction is heavily implicated. Mitochondria regulate immune cell function and survival by influencing their bioenergetic supply [36]. Metabolic demands are met through ATP production by glycolysis, the Krebs’ cycle, and, predominantly, oxidative phosphorylation. The degree to which immune cells utilise these pathways depends on the cell type, their activation state, and on substrate availability [37]. At rest, most immune cell types, with the notable exception of neutrophils, predominantly use oxidative phosphorylation to generate ATP necessary to perform housekeeping activities. However, on activation, immune cells place much greater reliance upon aerobic glycolysis (the Warburg effect), a process known as metabolic reprogramming. In addition to meeting bioenergetic needs, increased metabolites of the Krebs' cycle such as citrate and succinate play an important regulatory signalling role within these cells [38].

Neutrophils are short-lived innate immune cells that possess few mitochondria. While their metabolic needs are predominantly met by glycolysis, both in the sedentary and activated states, their effector functions include formation of neutrophil extracellular traps (NETs), phagocytosis and respiratory burst are under regulatory control by mitochondria [37].

B- and T-lymphocytes undergo metabolic reprogramming which both direct their differentiation into specific cell types and their functionality [39]. While glycolysis is generally upregulated, oxidative phosphorylation may be either up- or down-regulated depending upon the cell type [39,40]. Treg cells require fatty acid oxidation-fuelled oxidative phosphorylation for their effector functions [41–43]. These are discussed in more detail by Hortová-Kohoutková and colleagues [44].

The stimuli activating mononuclear cells may, at least in part, determine the source of the ATP. For instance, TLR-4 activation up-regulates glycolysis and reduces oxidative phosphorylation, while TLR-2 activation increases both glycolysis and oxidative phosphorylation [45]. In sepsis, in the presence of low glucose availability, monocytes up-regulate fatty acid oxidation and thus oxidative phosphorylation [46]. Macrophages and dendritic cells also reprogram their metabolism on activation though, again this depends upon specific cell type. Macrophages exist in two main phenotypes: M1 pro-inflammatory cells which function by up-regulation of glycolysis, pentose phosphate pathway and glutamine metabolism [47,48], and M2 anti-inflammatory cells that function via upregulation of oxidative phosphorylation driven by fatty acid oxidation and glutamine metabolism [49].

Studies implicate mitochondrial dysfunction in sepsis-induced leukocyte and organ dysfunction [50]. Impairment of electron transport chain complex production and activity, depolarisation of the mitochondrial membrane potential, increased ROS production and impaired biogenesis are described [51,52]. Although evidence underpinning mitochondrial dysfunction is consistent, the exact nature is conflicting and relates to heterogeneity in terms of timing, immune cell type, cell or animal model or patient and differing research methodologies [53,54]. Of note, functional recovery of mitochondria in peripheral blood mononuclear cells correlate with improved outcomes in septic patients [55].

## Endocrine changes during sepsis

### The normal stress response

**Figure 2:**
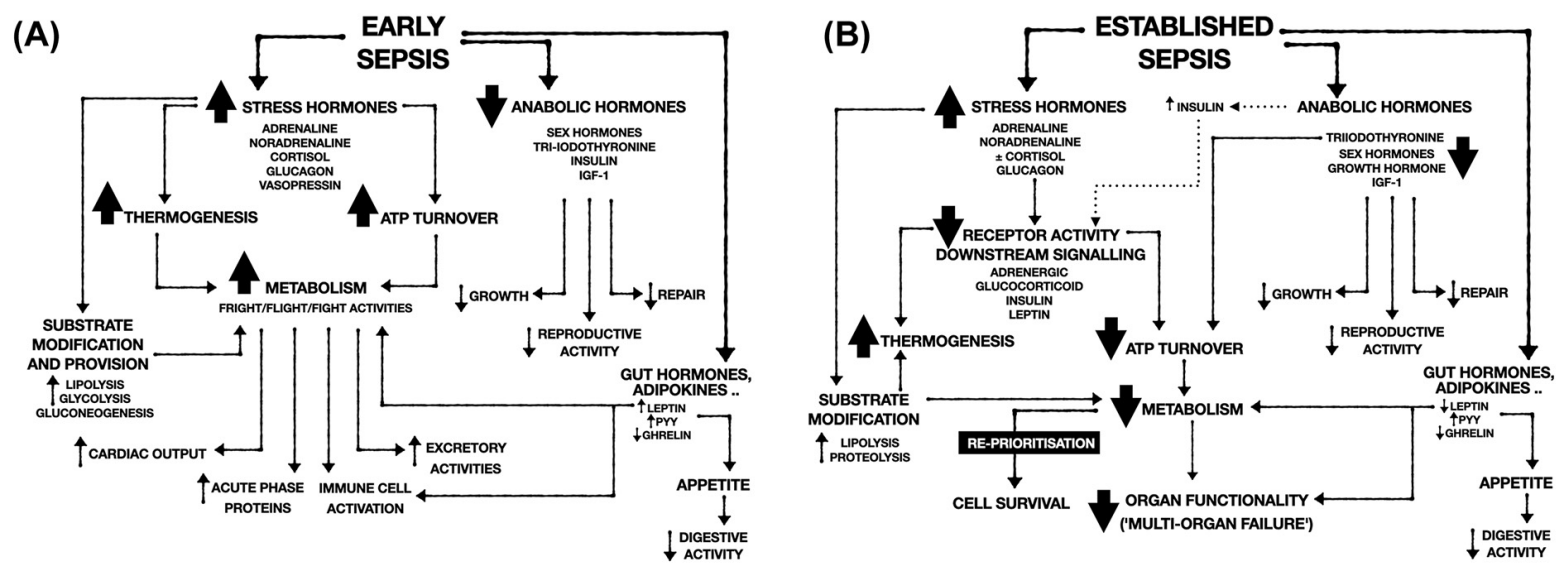
Modification of hormonal responses in early (A) and established (B) sepsis with downstream impact on immunity, metabolism, and organ functionality IGF-1 insulin growth factor-1; ATP adenosine triphosphate.

An important driver of metabolism and bioenergetic activity is the endocrine system. In response to any psychological or physical (e.g., exercise, trauma, and infection) stressor, there is widespread neurohormonal activation to adapt body behaviour and physiology to deal appropriately with the stressor. Production and secretion of stress hormones increase to modulate behaviour, whole body and organ blood flow, metabolic activity, substrate utilisation, and immune functionality (Figure 2).

The acute stress response initially involves rapid activation of the sympathetic-adreno-medullary system, with secretion of noradrenaline from sympathetic nerves, and adrenaline and noradrenaline from the adrenal medulla. Elevated catecholamine levels, acting through cell surface adrenergic receptors with downstream activation of the cyclic AMP (cAMP) pathway, heightens brain function, increases blood flow, prioritises flow to motor-active organs such as brain, heart and skeletal muscle, activates glycolysis and glycogenolysis to raise circulating glucose concentrations, stimulates lipolysis to increase free fatty acid concentrations as an alternative energy substrate, and induces thermogenesis to generate a febrile response.

Activation of the hypothalamus–pituitary–adrenal (HPA) axis leads to increased secretion of cortisol which, in turn, induces further catecholamine release, mobilizes energy stores through gluconeogenesis and glycogenolysis, and modulates the immune-inflammatory response. A rise in circulating glucagon stimulates gluconeogenesis and raises glycaemic levels to increase glucose availability. Vasopressin is released from the posterior pituitary gland, regulating blood pressure, blood volume and plasma osmolality. The renin–angiotensin–aldosterone system is also activated, encouraging salt and water retention.

The net effect of the stress response is an adaptation of behaviour towards increased arousal and focus, heightened analgesia but suppression of appetite and the reproductive axis. The physiological adaptations mobilise substrate (e.g., glycogenolysis to replete circulating glucose levels, free fatty acid and ketone bodies from ß-oxidation of fat, and lactate production by muscle), redirect oxygen and substrate towards stressed body locations, increase oxygen utilisation and detoxification processes, but inhibit digestion, growth, healing, and reproductive processes, and contain the immune/inflammatory response.

### The stress response in sepsis

The normal stress response is both adaptive and time-limited. However, severe and/or prolonged stress such as that seen during sepsis can lead to a state of dyshomeostasis and maladaptation (Figure 2). Many studies have been performed investigating specific hormonal perturbations such as critical illness-induced corticosteroid insufficiency, insulin resistance and adrenergic hyporesponsiveness, all of which are associated with worse outcomes. However, the endocrine system as a whole has been largely overlooked as a fundamental contributor to the integrated host response to sepsis and the development of organ dysfunction and immunosuppression.

The endocrine response during sepsis follows a distinct biphasic pattern. Acute changes are as described above, supporting the increased metabolic demands of the body [56], with a concurrent shutdown of less vital systems such as gonadal function and the digestive system. Catabolic pathways are up-regulated to drive essential cellular processes while anabolism is inhibited, most obviously witnessed clinically as insulin resistance [56], but also affecting other anabolic hormones such as growth hormone, insulin growth factor-1 and testosterone. In the later phase, after an undefined and variable period of critical illness ranging from hours to days, the hormonal profile alters substantially with loss of circadian rhythms, inappropriately low levels of vasopressin, adrenergic receptor downregulation, development of the ‘sick euthyroid syndrome’, and reduced adrenal responsiveness to adrenocorticotrophic hormone (ACTH), often despite high circulating cortisol levels [56–58]. The magnitude of these alterations, several of which will be discussed in more detail below, carry major prognostic implications [59,60].

Hypercortisolaemia results from both increased secretion of cortisol by proinflammatory cytokines, endothelin and other mediators [61], but also impaired clearance [62]. Normal diurnal variation is also lost [61]. Pro-inflammatory cytokines may also affect the number and binding affinity of glucocorticoid receptors [63,64]. The magnitude of rise and the response to ACTH reflect both severity of illness and prognosis [59].

The degree of rise in plasma catecholamine levels is also associated with increased mortality [65]. This may be a reflection of a greater stress response in more severely ill patients. However, persistently high levels of catecholamines have multiple potentially deleterious effects including altered splanchnic perfusion and impaired gut immunity, a marked increase in prothrombotic tendency, substrate modification towards fatty acid utilisation, stimulation of bacterial growth and virulence, and immune suppression [66]. There is also a concurrent down-regulation of adrenergic receptors and the adrenergic signalling pathway affecting vascular tone, myocardial contractility and immune functionality.

Insulin levels transiently fall during sepsis due to increased clearance rather than decreased secretion, increasing energy substrate availability [67]. However, the marked and prolonged rise in antagonistic catabolic hormones, particularly catecholamines, glucagon and cortisol [56,68,69], as well as down-regulation of insulin receptors [70,71], contribute to insulin resistance leading to hyperglycaemia and, eventually, hyperinsulinaemia [70,71]. The degree of insulin resistance is also associated with mortality and organ dysfunction [72].

The thyroid axis is affected during sepsis with decreased pituitary release of thyroid stimulating hormone (TSH) and inhibition of the peripheral conversion of thyroxine (T_4_) by 5-deiodinase to the much more metabolically active triiodothyronine (T_3_). High cortisol levels also inhibit this enzymatic conversion. Circulating T_3_ levels decrease while levels of the biologically inactive reverse T_3_ (rT_3_) increase; this phenomenon is known as the ‘sick euthyroid syndrome’. Changes in thyroid hormone levels also correlate with severity of illness [73]. Other abnormal aspects of the thyroid axis in sepsis include reduced concentrations of binding proteins, inhibition of hormone binding and changes in transport [74,75]. While TSH levels quickly decrease to the normal, pulsatile TSH secretion becomes suppressed. This correlates with suppressed TRH gene expression, implying a change in central regulation of the hypothalamic–pituitary–thyroid axis. As thyroid hormones are major regulators of metabolic processes, the net effect of the changes seen in sepsis is a reduction in energy expenditure and metabolic rate.

Hormonal changes during sepsis also modify eating behaviour. Appetite-inhibitory hormones such as the adipokine leptin and the gut hormone PYY initially rise in sepsis while ghrelin, an appetite-stimulatory peptide hormone released from the stomach, falls [76–78]. Whereas PYY remains elevated and ghrelin levels depressed over weeks [76], leptin levels subsequently fall [78,79]. The magnitude of the initial rise in leptin is associated with sepsis severity but, interestingly, survivors have higher levels than non-survivors [80,81]. This suggests that hyperleptinaemia may represent a host defence mechanism. Apart from appetite, leptin has multiple other roles, acting on metabolism, other endocrine functions, innate and adaptive immunity, and reproduction. As with other stress hormones, the situation is complicated further by the development of leptin resistance [77].

In addition to the endogenous stress response during both the acute and prolonged phases of sepsis, various stress hormones are often administered exogenously to critically ill patients. Not infrequently, these synthetic hormones are administered at supraphysiological doses. Examples include insulin to overcome insulin resistance and correct hyperglycaemia, catecholamines (noradrenaline, adrenaline, dobutamine) ± vasopressin ± angiotensin as circulatory support to increase blood pressure and/or cardiac output, and corticosteroids given for both their anti-inflammatory effects and for reversal of resistant hypotension by restoring vascular hyporeactivity.

## Impact of hormonal changes on mitochondrial function

Glucocorticoids and thyroid hormones regulate metabolism through modifying mitochondrial function and biogenesis. Their receptors interact with mitochondrial and nuclear response elements affecting transcription factors and thus expression of nuclear- and mitochondrial-encoded genes [82]. These hormones also have rapid non-genomic effects on mitochondria involving cytoplasmic kinase signaling pathways [83]. These pathways result in alterations in the structure and function of key mitochondrial components including those of the electron transport chain (Table 1).

**Table 1:** Endocrine-induced effects changes in mitochondrial function and immune cell function during the acute and established phase of sepsis

Hormone	Endocrine changes	Mitochondrial changes	Immune changes
**Cortisol**	**Acute:** ↑	**Acute:** ↑O_2_ and energy substrate availability; ↑aerobic glycolysis; ↑biogenesis; ↓apoptosis; ↓UCP-1 and UCP-3 but ↑UCP-2.	**Innate:** ↑↓ immune function including cell differentiation, phagocytosis and cytokine release.
	**Established:** ↑↓ with loss of diurnal variation. Often ↓response to exogenous stimulation.	**Established:** ↓biogenesis; ↑apoptosis; ↑ROS.	**Adaptive:** ↓lymphocyte activation but ↑apoptosis; ↓cytokines and chemokines; ↑Th_2_ and Treg cell expression over Th_1_ and Th_17_ cells.
**Catecholamines**	**Acute:** ↑	**Acute:** ↑O_2_ and energy substrate availability; ↑aerobic glycolysis; ↑Ca^2+^ accumulation; ↑ETC expression and activity; ↑biogenesis; ↑apoptosis; ↑ROS in skeletal muscles but ↓ROS in immune cells.	↓gut immunity; ↑bacterial growth and virulence; ↑immune suppression.
	**Established:** ↑↓ but increased hypo-responsiveness.	**Established:** ↓Ca^2+^ influx; ↓ETC function; ↓ O_2_ consumption and ↓ oxidative phosphorylation.	**Innate:** α-ARs activation ↑inflammation; β_2_-AR activation ↓inflammation including chemotaxis, phagocytosis and ROS for respiratory burst.
			**Adaptive:** β_2_-AR activation ↓T-cell proliferation but ↑Th2 polarisation.
**Thyroid hormones**	**Acute:** ↑ but soon after ↓TSH; ↓T_4_ to T_3_ conversion; ↓T_3;_ ↑rT_3_ (sick euthyroid syndrome).	**Acute:** ↑ETC expression and activity; T_3_ ↑mitochondrial mass but ↓efficiency of ATP production; ↑↓biogenesis; ↑↓apoptosis; ↑UCP; hypothyroidism ↓proton leak.	**Innate:** ↑↓ chemotaxis, phagocytosis and respiratory burst. Sick euthyroid syndrome ↓immune function; ↑monocyte differentiation to DCs rather than macrophages.
	**Established:** TSH normalises but loses pulsatility; ↓TRH; ↓T_3_.	**Established:** ↑biogenesis	**Adaptive:** ↑↓ lymphocyte proliferation and apoptosis, and B-cell antibody production
**Insulin**	**Acute:** ↓	**Acute:** ↑O_2_ and energy substrate availability; ↑aerobic glycolysis; ↑biogenesis; ↑ROS	**Innate:** ↓respiratory burst and NET formation in neutrophils; ↓ proinflammatory cytokines.
	**Established:** ↑ but also ↑insulin resistance.	**Established:** ↑↓ETC function and ATP production; ↓apoptosis.	**Adaptive:** ↑lymphoid cell lineage expression; ↑T-cell proliferation, differentiation, and effector functions.
**Glucagon**	**Acute:** ↑	**Acute:** ↑O_2_ and energy substrate availability; ↑aerobic glycolysis; ↑ETC expression and activity and ↑ATP; ↓apoptosis.	**Innate:** ↓chemotaxis and respiratory burst; ↑↓ neutrophil numbers.
	**Established:** ↑	**Established:** ↓biogenesis	**Adaptive:** ↓T-cell proliferation, differentiation, and effector functions.
**Leptin**	**Acute:** ↑	**Acute:** ↓↑apoptosis; ↑ROS.	**Innate:** ↑cytotoxicity of NK cells; ↑activation of granulocytes, DCs and macrophages.
	**Established:** ↓	**Established:** ↑biogenesis in BAT.	**Adaptive:** ↓T-cell proliferation and responsiveness; ↓Th cell differentiation; ↑Treg cell proliferation; ↓B-cell proliferation but ↑apoptosis.

Abbreviations: AR, adrenergic receptor; ATP, adenosine triphosphate; BAT, brown adipose tissue; DC, dendritic cell; ETC, electron transport chain; NK, natural killer cell; ROS, reactive oxygen species; Th, T-helper cell; Treg, regulatory T-cell; UCP, uncoupling protein.

The combination of early rises in cortisol, catecholamines, and glucagon during sepsis in conjunction with an initial decrease in insulin rapidly impacts upon bioenergetics and metabolic activity. This initially includes increased oxygen and energy substrate availability as well as accelerated aerobic glycolysis to support increased tissue bioenergetic demands [70,84]. Insulin resistance and hyporesponsiveness to glucocorticoids during the prolonged phase of sepsis may, however, result in an inability to meet metabolic requirements. Although the classic thyroid hormones (T_4_ and T_3_) have been widely studied, little is known about the effects on mitochondria of rT_3_. In chickens rT_3_ suppressed levels of free fatty acids in response to stressors [85]. The conversion switch from free T_3_ to metabolically inactive or even suppressive rT_3_ may serve as an adaptive coping mechanism to conserve energy.

### Oxidative phosphorylation

*In vivo* and *in vitro* studies demonstrate that glucocorticoids affect mitochondrial function of kidney, brain, and muscle in a biphasic manner [86]. Short-term and/or low levels appear protective, inducing calcium accumulation and increasing both expression and activity of electron transport chain components. However, long-term exposure and/or high concentrations cause mitochondrial dysfunction with inhibition of calcium influx and holding capacity and decreased activity of the respiratory chain, ultimately resulting in decreased oxidative ATP production.

Thyroid hormones rapidly enhance mitochondrial respiration and ATP generation associated with the expression of electron transport chain components and accelerated translocation of ATP into the cytosol [87,88]. Liver mitochondria isolated from hypothyroid rats had lower resting rates of oxygen consumption [89]. Studies in sepsis are however limited. Septic mice had impaired diaphragm mitochondrial numbers and activity with a decrease in maximal respiration alongside a fall in serum T_4_ and a decrease in thyroid hormone signalling [90]. In this model, treatment with thyroid hormones at the onset of sepsis protected mitochondrial parameters but did not impact on survival. By contrast, T_3_ replacement in patients with established sepsis showed no improvement in respiratory muscle function [91].

Mitochondrial effects of catecholamines are variable and depend on cell type, timing and dose. In early sepsis, adrenaline and noradrenaline will rapidly increase respiratory enzyme activity, aerobic respiration and ATP production in liver [92–94], with reduced mitochondrial enzyme function following depletion of noradrenaline or receptor blockade [95,96]. On the other hand, reduced oxygen consumption and spare respiratory capacity (SRC) was seen in both primary human monocytes and PBMCs upon direct exposure to noradrenaline and adrenaline [97–99]. This may represent a functional metabolic switch in these immune cells. However, these noradrenaline- and adrenaline-trained cells did show an increase in oxidative phosphorylation after 6 days. Conflicting results were found by the same group in a porcine model of faecal peritonitis, with either no effect or enhancement of liver mitochondrial respiration by noradrenaline [100,101].

A wide range of studies have shown stimulatory effects of glucagon on mitochondrial respiration, the protonmotive force, electron chain complex function and a rise in ATP in liver, brain, and adipose tissue during a period of increased energy demand [102–107]. Glucagon enhancement of mitochondrial function may relate to a rise in cAMP levels or increase in mitochondrial calcium retention [108].

Mitochondrial dysfunction has been implicated as contributory towards insulin resistance [109], but the importance of insulin signalling for normal mitochondrial function has also been demonstrated in multiple tissues. Insulin is pivotal for mitochondrial function and usually stimulates respiration, enzyme activity and ATP production is a variety of tissues [110,111]. Both insulin deficiency and insulin resistance as seen during later phases of sepsis, have been associated with decreased respiration and ATP production. A more recent study also indicated biphasic insulin induced effects, with acute exposure leading to increased biogenesis and enzyme activity, while chronic exposure had variable effects [112].

### Mitochondrial biogenesis

Turnover of new mitochondria (biogenesis) is also influenced by hormonal changes. Low and/or short-term exposure to corticosteroids increased mitochondrial biogenesis and mitochondrial DNA content [113]. Similar effects are reported with thyroid hormones, catecholamines, and insulin [87,92,110]. Corticosteroids and thyroid hormones have direct and indirect effects on co-activators and transcription factors of biogenesis, affecting nuclear and mitochondrial-encoded genes. Thyroid hormones also modulate chromatin structure of genes, thereby affecting gene expression. However, the regulation of mitochondrial biogenesis by thyroid hormones appears to be tissue-specific as no or opposing effects were observed in heart tissue [114]. Stimulation of β-adrenergic receptors by adrenaline and noradrenaline promoted mitochondrial biogenesis and increased mitochondrial content non-genomically [115]. As with thyroid hormones, catecholamine-driven stimulation of mitochondrial biogenesis via the transcription coactivator, peroxisome proliferator-activated receptor-γ coactivator-1α (PGC-1α) also appears to be tissue specific [116]. The insulin-mediated increase in mitochondrial function, with mTOR and FOXO acting as downstream effectors, may be due to increased expression of electron transport chain complexes; insulin deficiency and resistance both decrease mitochondrial biogenesis [110]. The subsequent increase in mitochondrial mass may be responsible for elevations in resting metabolic rate [117].

By contrast, long-term or high dose exposure to corticosteroids results in abnormal regulation of mitochondrial biogenesis, especially in skeletal muscle *in vivo* and *in vitro* [86]. In addition to its rapid-onset effects on mitochondria, T_3_ is also a long-term regulator of mitochondrial biogenesis via PGC-1α, increasing mitochondrial mass [118]. Lower circulating levels of this hormone during sepsis could act as a counterbalance. Long-term glucagon exposure also suppresses mitochondrial biogenesis via FOXO1 and regulation of sirtuins [107,119]. The effects of leptin on mitochondrial biogenesis are conflicting, but stimulation via PGC-1α may occur in brown adipose tissue [120].

### (Un)coupling and ROS

Glucocorticoids inhibited the activity of the uncoupling proteins UCP-1 and UCP-3 in brown adipose tissue [121] thereby increasing mitochondrial membrane potential, but up-regulated UCP-2 in microvascular endothelial cells [122]. No effects were seen in skeletal muscle [117]. Induction of uncoupling is regulated by both glucocorticoid- and mineralocorticoid receptors, to which these hormones bind with varying affinity. Although uncoupling increases oxygen consumption while decreasing mitochondrial membrane potential and energy substrate availability, it could also be an adaptive mechanism to limit harmful production of mitochondrial ROS [122]. Chronic exposure to corticosteroids is, however, related to an increase in ROS production [86].

Despite increased respiration and ATP generation, T_3_ reduces the efficiency of these processes while hypothyroid states reduce proton leak [89]. In brown adipose tissue, UCP-1 up-regulation appears responsible but lower basal proton leak in mitochondria from hypothyroid rats in other tissues is not yet fully understood. Possible mechanisms include induction of UCP-2 or UCP-3, or changes in phospholipid composition of the mitochondrial membrane [87]. Overall, thyroid hormones are associated with increased ROS production and potential damage due to augmented oxidative metabolism and decreased antioxidant protection; hypothyroid states on the other hand decrease ROS [123].

Catecholamines also increase ROS production and are associated with oxidative damage to liver [92,124], and skeletal muscle [115]. Surprisingly, the increase in ROS in skeletal muscle occurred in conjunction with a reduction in mitochondrial membrane potential. The respiratory control ratio (RCR) increased with noradrenaline and adrenaline use in endotoxaemic models [93,101], yet respiratory efficiency was impaired [125]. Although mitochondria are not the only source of ROS in phagocytic cells, noradrenaline reduced ROS production in stimulated primary monocytes as well as in endotoxin-stimulated neutrophils but suppressed the respiratory burst in non-LPS challenged neutrophils [97].

An important role for glucagon in the regulation of thermogenic regulation in brown fat has been shown by induction of thermogenic genes and by increasing nucleotide binding (GDP) [126,127]. Glucagon treatment induced a coupling defect in liver and skeletal muscle mitochondria [128], while the RCR did not change in brain mitochondria [103]. Insulin deprivation and resistance are characterised by declined coupling efficiency concurrent with excessive ROS and oxidative damage [110]. The lack of sufficient antioxidant defences normally enhanced by insulin, and glucose-mediated ROS production may be contributory [129–131].

Leptin has also been shown to increase mitochondrial superoxide production by increasing fatty acid oxidation [132].

### Apoptosis

Although endocrine induced effects on apoptosis have been widely described, it must be noted that apoptosis is regulated by both intrinsic mitochondrial pathways and extrinsic non-mitochondrial pathways. Glucocorticoids are well-known regulators of apoptosis during lymphocyte maturation, but mixed effects have been found depending again on duration of exposure and concentration. Acute and/or low doses of glucocorticoids protect mitochondria and prevent programmed cell death, while chronic and/or long-term exposure increase apoptosis [86].

Thyroid hormones play a role in the initiation of apoptosis, eliminating unwanted cells, including T-lymphocytes. This may be mediated in part by increasing cytosolic calcium content, opening of the mitochondrial permeability transition pore (mPTP) and modulation of pro- an anti-apoptotic proteins, in addition to direct genomic effects [133]. By contrast, anti-apoptotic effects have also been described in cancer cell lines, neurons, fibroblasts and myocardial cells, down-regulating p53, pro-apoptotic proteins, and caspases [134–137].

Intrinsic pro-apoptotic effects of catecholamines acting via the β-adrenergic receptor have been found in various cell types. Noradrenaline may exert these effects via different pathways including ROS production, inhibition of the PI3K/Akt survival pathway and caspase activation [138,139]. Generally similar effects have been found for adrenaline.

Knowledge of the impact of other hormones on the mitochondrial apoptotic pathway is more limited. Chronic insulin exposure decreased cytochrome *C* expression, suggesting an antiapoptotic effect [112]. Glucagon delayed the onset of mPTP opening, protecting cells from apoptosis after ischaemia-reperfusion, and potentially acts via the cAMP/PKA pathway [140,141]. The effects of leptin are conflicting, with promotion of apoptosis in adipose tissue and heart via calcium-induced mPTP opening [142,143], yet anti-apoptotic effects on the heart, immune and neuronal cells [144–146].

### Other mitochondrial changes

Other long-term glucocorticoid effects on mitochondria include structural abnormalities with mitochondrial damage due to induced hyperglycaemia [147]. Strict glycaemic control with insulin therapy prevented ultrastructural and functional abnormalities of liver mitochondria [131]. The increased cellular energy demands during stress with associated increases in mitochondrial ROS production can damage mitochondria when antioxidant defences are overwhelmed. Mitophagy is a quality control mechanism that can be induced by T_3_ to limit ROS-induced damage [148]. Insulin deprivation increases markers of mitophagy [149]. In rats, isoprenaline, a synthetic catecholamine, promoted cardiac mitochondrial dysfunction by opening the mPTP and increasing mitochondrial membrane swelling, while noradrenaline protected skeletal muscle mitochondria from propofol-induced dysfunction [150]. This may be especially relevant to septic patients who are sedated. Mitochondria are protected by glucagon by changes in the disposition of the inner mitochondrial membrane [151]. Mice lacking both insulin and IGF-1 receptors showed morphological changes in cardiac tissue preceded by down-regulation of genes encoding for electron transport chain and fatty acid β-oxidation pathways within mitochondria and altered expression of contractile proteins [152].

## Impact of hormonal changes on immune cell function

We should start with the important caveat that much of the current literature is based upon *ex vivo* or *in vitro* incubation of isolated cells or cell lines with hormones, with or without stimulation by lipopolysaccharide, and often at concentrations markedly higher than those measured *in vivo* in the septic patient [97]. Furthermore, the cells are isolated from their *in vivo* milieu; influences from other immune cells, circulating mediators and other hormones within plasma, and endothelial interactions are removed. As a consequence, the literature is often inconsistent and direct translation to the *in vivo* situation in the septic patient is uncertain.

While stress hormones generally induce immune suppression [153] this is not straightforward. Even cortisol, generally considered the archetypal anti-inflammatory stress hormone, can be pro-inflammatory under certain conditions. The type of immunomodulation depends not only on circulating levels and duration of elevation, but also the cell type and the type of receptor being activated. Catecholamines, glucagon and insulin induce non-genomic signals, while mechanisms underlying glucocorticoid and thyroid hormone activity also include genomic pathways regulating gene transcription [82] (Table 1). An important question is whether the effects of these hormones on the immune system are additive, or whether some of the signalling pathways become saturated or unresponsive.

Another important point to make is that the native host response is heavily modified by exogenous administration of hormones that are frequently used in the management of septic patients, and often at supraphysiological doses. Common examples include catecholamines, vasopressin or its analogues, corticosteroids and insulin. The stress response and immune function are also modified by other routine interventions, for example the use of immunomodulating sedative drugs [154] and a decrease in sympathetic activity due to the patient being asleep.

### The innate immune system

Despite their generally anti-inflammatory effects, glucocorticoids appear to act in a biphasic manner. Low doses of endogenous glucocorticoids, or exposure to this hormone without an additional inflammatory stimulus, can enhance pathways involved in the innate immune response by up-regulating PRRs, cytokine receptors and complement factors. This includes aiding differentiation of macrophages, promoting phagocytosis of apoptotic cells and debris by monocytes and macrophages, and anti-inflammatory cytokine secretion [155]. Expression of pro- and anti-inflammatory genes are regulated via NF-κB and AP-1 or by post-translational protein modification [155]. In contrast, glucocorticoids exert anti-inflammatory effects by inhibiting expression and secretion of pro-inflammatory cytokines and chemokines, impairing phagocytosis in macrophages, increasing apoptosis of neutrophils, basophils and eosinophils, and decreasing antigen presentation and co-stimulation by dendritic cells which will ultimately affect the adaptive immune system [155–157].

The effects of catecholamines on the immune system are also complex [158]. Catecholamines bind to α- and β-adrenergic receptors with variable affinity depending on the dose and type of catecholamine; they also exert a range of effects that depend on receptor subclass and location. Temporal changes in receptor density and downstream signalling are poorly characterised at present. α-adrenergic receptors have predominantly pro-inflammatory actions by activating NF-κB and increasing pro-inflammatory cytokines *in vitro*. By contrast, β_2_-adrenergic receptor activation via cAMP-PKA signal transduction inhibits NF-κB and reduces production of pro-inflammatory cytokines, while increasing anti-inflammatory cytokines such as IL-10. β_2_-AR activation also inhibits chemotaxis, phagocytosis and the respiratory burst in neutrophils, phagocytosis in macrophages *in vitro* and reduces NK-cell cytotoxicity.

Thyroid hormones play an essential role in the innate immune response at both genomic and non-genomic levels. Both hyper- and hypothyroidism affect immune cell functionality, including chemotaxis, cytokine release, phagocytosis, and bacterial killing. Potentially comparable to the ‘sick euthyroid syndrome’ seen during sepsis is hypothyroidism. This state is generally associated with a decreased immune response as evidenced by reduced migration and chemotaxis ultimately affecting mortality [159]. Some of these innate immune functions were restored after supplementation. However, evidence is conflicting as increased release of pro-inflammatory markers and mixed effects on respiratory burst activity have also been reported [160]. Thyroid hormones decrease migration of neutrophils but have also been shown to increase neutrophil cell numbers and bacterial killing by increasing respiratory burst activity [159]. Physiological levels of T_3_ are essential for NK-cell activity [159]. Thyroid hormones also favour monocyte differentiation into dendritic cells rather than macrophages [161]. Increased phagocytosis and respiratory burst with decreased M2 polarisation have been observed in macrophages [159,161,162]. In dendritic cells, T_3_ increased cell maturation, activation, viability, migration, and antigen presenting cell (APC) function [161,163].

Insulin seems to favour the adaptive immune response, shifting differentiation of bone marrow progenitor cells towards a lymphoid cell lineage [164]. Other anti-inflammatory effects of insulin include a reduction in respiratory burst and NET formation in neutrophils and reduced pro-inflammatory cytokine production [165]. In patients with Type 2 diabetes, insulin reduced TLR transcription after LPS stimulation [166–168]. These effects are mediated by multiple mechanisms, including glucose toxicity and related oxidative damage [169], inhibition of FOXO1 transcription factor via activation of the P13K-Akt signalling pathway [167], indirect regulation of NRF2 [169], suppression of NF-κB, and/or modulation of autophagy [170–172]. As insulin levels initially decrease during the early phase of sepsis, immunomodulating effects may be mild.

Elevated levels of glucagon may also contribute to dysregulation of innate immune cells. Reduced bacterial killing and adaptive immune activation were seen after exposure to high concentrations of glucagon, as evidenced by an impaired respiratory burst, reduced chemotaxis and neutrophil accumulation [173], a shift in gene expression of pro- and anti-inflammatory cytokines in monocytes [174], and reduced numbers and activity of NK-cells [175–177]. However, conflicting reports show increased superoxide production in neutrophils and improved cell survival after blockade of the glucagon receptor [178].

Leptin increases cytotoxicity of NK cells and promotes activation of granulocytes, dendritic cells and macrophages with release of proinflammatory cytokines. On the other hand, leptin deficiency, as seen during prolonged sepsis, increases susceptibility to infections [77].

### The adaptive immune system

Glucocorticoids regulate adaptive immunity by inhibiting lymphocyte activation and promoting lymphocyte apoptosis, events also observed in sepsis [156,157]. At high concentrations, B- and T-cell production is also inhibited [157]. Glucocorticoids inhibit pro-inflammatory genes involved in adaptive immunity and also dampen signals downstream of PRRs, cytokine receptors and Fcɛ receptors. They inhibit expression of chemokines and adhesion molecules that curtail inflammation and reduce leukocyte recruitment [179–182], directly suppress CD4^+^ T-cell activation and favour differentiation of T-cells into Th2 and Treg cells over Th1 and Th17 cells.

Similar to the abovementioned anti-inflammatory effects of glucocorticoids, β_2_-adrenergic receptor activation by catecholamines also affects the adaptive immune response by suppressing T-cell proliferation and shifting differentiation of Th cells towards Th2 polarisation. This subsequently reduces the production of IFN-γ by Th1 cells and the ability to fight intracellular bacterial infections [158]. As with their effects on innate immune cells, α-adrenergic receptor activation increases the production of pro-inflammatory cytokines, while β_2_-adrenergic receptor activation favours production of anti-inflammatory cytokines [158]. Of note, elevated levels of catecholamines have been reported up to two years after critical illness [183]; this is associated with immunosuppressive effects that persist long after hospital admission and increase susceptibility to secondary infection and risk of hospital re-admission.

Findings on thyroid hormone-induced effects on humoral and cell-mediated immune immunity are less well known and conflicting, with studies both indicating an increase and decrease in lymphocyte proliferation and apoptosis and B-cell antibody production [160,184–187]. Hypothyroidism has mainly been associated with a decreased immune response as indicated by decreased lymphocyte proliferation, but increased release of pro-inflammatory markers. Supplementation subsequently reversed some of these effects. Effects on other aspects of the adaptive immune system including antibody production are not consistent [160].

Although insulin favours the differentiation of progenitor cells towards the lymphoid cell lineage, it also increases T-cell function by stimulating proliferation, differentiation and effector function. This is regulated by changes in metabolism and activation of the P13K-Akt-mTOR pathway [188,189]. By contrast, insulin favoured polarisation of lymphocytes into the Th2 anti-inflammatory phenotype [190]. Insulin did not however induce substantial changes in B-cells [191]. Reduced accumulation, proliferation and function of T-cells has been reported with glucagon treatment [192].

Leptin induces T-lymphocyte proliferation and responsiveness, increasing Th cell differentiation but decreasing Treg cell proliferation. It also increases proliferation and has antiapoptotic effects on B-lymphocytes [77].

## Conclusion

Sepsis is a complicated syndrome with various interlinked bodily systems that are affected in a time-dependent manner, making it difficult to translate findings to a clinical setting. We do appreciate that the current review focuses on a simplified selection of stress and metabolic hormones, making that the overall picture is even more complicated. Despite this, it is evident that immune cell function depends on mitochondrial function, and that the hormones discussed affect both immune cell and mitochondrial function which could significantly contribute to mortality in sepsis [59,60,193]. Supplementing endogenous changes with exogenous administration of, e.g., insulin, catecholamines, and hydrocortisone [193] could therefore be detrimental for patients in the long run. However, other improved treatment strategies are currently lacking. Additionally, there is still some controversy to be found in the literature and limited knowledge on underlying mechanisms. Variations might be largely due to differences in study methodologies. This includes differences in tissues and cells studied, exposure duration and timing, septic source or stimulus, dose and formulation of hormones used. Further studies are required to fully elucidate how each of these hormones may affect the immune system and mitochondria, especially studies with clinically relevant concentrations in those cells of the innate and adaptive immune system, and more importantly how these hormones work in unison to mediate some of the commonly seen changes in sepsis.

## Open Access

Open access for this article was enabled by the participation of University College London in an all-inclusive *Read & Publish* agreement with Portland Press and the Biochemical Society under a transformative agreement with JISC.

## Abbreviations

ACTH: adrenocorticotrophic hormone
APC: antigen presenting cell
BAT: brown adipose tissue
HPA: hypothalamus–pituitary–adrenal
mPTP: mitochondrial permeability transition pore
PAMP: pathogen-associated molecular pattern
PD-1: programmed cell death-1
PRR: pattern recognition receptor
RCR: respiratory control ratio
